# Safety Assessment of Polypyrrole Nanoparticles and Spray-Coated Textiles

**DOI:** 10.3390/nano11081991

**Published:** 2021-08-03

**Authors:** Rossella Bengalli, Luisa Fiandra, Claudia Vineis, Diego Omar Sanchez-Ramirez, Nuno G. Azoia, Alessio Varesano, Paride Mantecca

**Affiliations:** 1 POLARIS Research Center, Department of Earth end Environmental Sciences, University of Milano-Bicocca, Piazza della Scienza 1, 20126 Milano, Italy; paride.mantecca@unimib.it; 2 Department of Biotechnology and Biosciences, University of Milano-Bicocca, Piazza della Scienza 2, 20126 Milano, Italy; luisa.fiandra@unimib.it; 3 National Research Council-Institute of Intelligent Industrial Technologies and Systems for Advanced Manufacturing (CNR STIIMA), Corso Giuseppe Pella 16, 13900 Biella, Italy; Claudia.Vineis@stiima.cnr.it (C.V.); DiegoOmar.SanchezRamirez@stiima.cnr.it (D.O.S.-R.); Alessio.Varesano@stiima.cnr.it (A.V.); 4 CeNTI-Centre for Nanotechnology and Smart Materials, Rua Fernando Mesquita, 2785, 4760-034 Vila Nova de Famalicão, Portugal; nazoia@centi.pt

**Keywords:** nanoparticle-coated textiles, nanosafety, polypyrrole nanoparticles, air–blood barrier, skin exposure

## Abstract

Polypyrrole (PPy) nanoparticles (NPs) are used for the coating of materials, such as textiles, with biomedical applications, including wound care and tissue engineering, but they are also promising antibacterial agents. In this work, PPy NPs were used for the spray-coating of textiles with antimicrobial properties. The functional properties of the materials were verified, and their safety was evaluated. Two main exposure scenarios for humans were identified: inhalation of PPy NPs during spray (manufacturing) and direct skin contact with NPs-coated fabrics (use). Thus, the toxicity properties of PPy NPs and PPy-coated textiles were assessed by using in vitro models representative of the lung and the skin. The results from the materials’ characterization showed the stability of both the PPy NP suspension and the textile coating, even after washing cycles and extraction in artificial sweat. Data from an in vitro model of the air–blood barrier showed the low toxicity of these NPs, with no alteration of cell viability and functionality observed. The skin toxicity of PPy NPs and the coated textiles was assessed on a reconstructed human epidermis model following OECD 431 and 439 guidelines. PPy NPs proved to be non-corrosive at the tested conditions, as well as non-irritant after extraction in artificial sweat at two different pH conditions. The obtained data suggest that PPy NPs are safe NMs in applications for textile coating.

## 1. Introduction

The production of nano-enabled products (NEPs) and in particular of nanocoated textiles, is growing fast due to the multiple applications for these products such as, for example, antipollutant, self-cleaning, antireflective, antibacterial, photocatalytic, and conductive agents [1]. Moreover, textiles functionalized with nanoparticles (NPs) have found different employments in tissue repair and engineering [2] and are known as good candidates for the grafting with enzymes [3].

Furthermore, the COVID-19 pandemic has intensified the demand for producing more efficient materials against microbes and viruses for the coating of surfaces, including textiles and face masks [4]. Additionally, the high costs and deaths due to nosocomial infections push industries to develop antibacterial materials with high efficiency and low cost. In particular, microbial resistance to antibiotics is indeed an important issue that draws attention to the synthesis and market displacement of new products, against resistant pathogens [5,6]. As expected, in parallel, the release of nanomaterials (NMs) from enabled goods and during manufacturing is a matter of concern from an environmental and human health standpoint [7].

Silver NPs (Ag-NPs) and other metal oxide NPs (MeO-NPs), such as copper and zinc oxide and titania NPs, are often used for the coating of textiles with medical purposes, thanks to their promising and efficient antimicrobial activity [8]. However, these NMs used for the enabling of products could be hazardous and that is mainly due to the high solubility of these NPs, which in turn causes ion release with consequent undesired toxicity in non-target areas, e.g., mammalian cells or aquatic systems.

Polymeric particles, such as polypyrrole (PPy) NPs, seem to be good alternative candidates for textiles coating to metal-based and MeO-NPs, which however still retain pre-eminence for their high antibacterial properties. PPy is a conjugated polymer produced by chemical oxidative polymerization. The antibacterial properties of PPy, which are due to the positive charge of the NPs along the backbone chain of polypyrrole, have been reported, especially for the coating of textiles with antimicrobial properties [9]. Consequently, strong electrostatic interaction is established with negatively charged surfaces, such as the cell wall of Gram-negative bacteria (e.g., *Escherichia coli*) [10,11]. The PPy positive charges have an impact on the bacteria cell wall, which is disrupted after exposure to PPy-coated textiles. In a previous work, indeed it has been shown, by scanning electron microscopy (SEM) analysis, that the *E. coli* cell wall was broken when in contact with PPy-coated fabrics and the bacteria appeared flattened, due to the leakage of intracellular material. On the contrary, bacteria survived on the uncoated fabrics [9].

PPy is also used in different biomedical applications due to its biocompatibility [12] and has received a lot of attention in bioelectronic applications due to its properties, such as high conductivity, stability, and compatibility with biological components [13,14,15]. Biocompatible conductive polymers are also promising fillers for conductive hydrogels. Thanks to their good conductivity and flexibility, they form a well-connected electric path due to a uniform distribution of the hydrophilic NPs in hydrogel networks. These materials exhibits long-term adhesiveness and compatibility to different substrates, such as glass, plastic, steel, and also human skin and rat muscle [16]. Thus, conductive polymers could find many applications in medical therapies, such as tissue repair by electrical stimulation or implantation of biosensors, but could also be incorporated into microcapsules for the control of drug delivery [17].

However, different degrees of binding of the NPs to the selected surface are responsible for their possible release and, consequently, human exposure. As far as human beings are concerned, two of the main routes of exposure to such NMs and the respective NEPs are inhalation and skin contact. Inhalation occurs especially during the production of NPs and during coating processes, such as spray, while skin exposure happens through direct contact with NEPs used for example in textiles or creams/lotions.

In a safe-by-design perspective, health and safety concerns related to an NEP, especially in the case of the application of NMs onto fabrics, should take into account all the life cycle steps of the material itself [18], such as production, manufacturing process, handling, end use, and disposal. In this work, two main exposure scenarios to NMs were identified: (i) inhalation by workers during the coating process of the textiles; (ii) direct skin contact exposure to NPs-coated fabrics for end users.

In parallel to the production and characterization of the physical, chemical, and antibacterial properties of the PPy and coated textiles, the aim of this work is the safety assessment of the new NMs using in vitro models representative of the lung (alveolar cells, air–blood barrier model) and the skin (Balb/3T3 fibroblasts; EpiDerm™).

The results obtained suggest that the use of these NMs in applications for textile coating is safe.

## 2. Materials and Methods

### 2.1. Polypyrrole Synthesis

The synthesis of PPy nanoparticles follows the procedure described in literature [19].

Briefly, a solution of 4.1 wt.% of APS (ammonium persulfate ≥98%, Sigma Aldrich, Milano, Italy), and 14.6 wt.% of PSS (polystyrene sulfate, Sigma Aldrich) was prepared in distilled water. Pyrrole (monomer, 97%, Sigma Aldrich) was added at a final concentration of 4.1 wt.%. The solution was maintained for 7 days at room temperature. Then the polymerization was stopped by adding 5 N sodium hydroxide (NaOH, Sigma Aldrich) solution to the dispersion until a pH value over 5.0 was reached.

### 2.2. PPy NPs Spray Deposition on Fabrics

Before deposition on fabric (100% cotton), the PPy solution was diluted in a 1:4 ratio with distilled water. Ultrasonic spray nozzles (output frequency of 58 kHz) were used for the deposition, where the ultrasonic probe of the nozzle improves the atomization of the solution, and compressed air (2 bar) is used to improve the spray efficiency. A setup of three nozzles was used, meaning each fabric sample was subjected to three spray depositions. The solution was fed to each nozzle at a flow rate of 50 mL/min, by a peristaltic pump. The distance from the nozzle to the fabrics was 40 cm, and during the deposition, the fabric was moving forward with a constant speed of 2 m/min, by means of a conveyor belt. To ensure proper drying of the fabrics, prior to the spray deposition, each sample was dried in an oven, at 100 °C, for 10 min.

### 2.3. NPs and NPs-Coated Textiles’ Characterization

PPy NPs were morphologically characterized by transmission electron microscopy (TEM). Briefly, 10 µL of PPy NP suspension diluted (100 µg/mL) in sterile mQ water was dropped into a Formvar^®^-coated 200-mesh copper grid. After overnight drying, grids were observed by means of a Jeol JEM 2100Plus (JEOL, Tokyo, Japan) TEM, operating with an acceleration voltage of 200 kV and equipped with a 9MPx complementary metal oxide semiconductor (CMOS) Gatan Rio9 (Gatan, Pleasanton, CA, USA) digital camera.

The PPy NPs’ (final concentration 100 µg/mL) size distribution was evaluated through DLS (dynamic light scattering) analyses by using a Malvern Zetasizer (Malvern, UK). The NPs’ hydrodynamic behavior was assessed in different media: Milli-Q (mQ) sterile water, DMEM (Gibco, Life Technologies, Monza, Italy) medium with 1% fetal bovine serum (FBS, Gibco), and Opti-MEM (Gibco) medium supplemented with 1% FBS. The following media were used for the in vitro exposure experiments: NPs suspended in water were used for the skin corrosion test on a reconstructed epidermis model, NPs in Opti-MEM 1% FBS were prepared for in vitro experiments with lung cells, and NPs in DMEM 1% FBS medium were used for Balb/3T3 fibroblast exposure to PPy NPs.

PPy-coated fabrics were characterized by an EVO10 Scanning Electron Microscope (Carl Zeiss Microscopy GmbH, Oberkochen, Germany) with an acceleration voltage of 20 kV at about 13 mm working distance. Uncoated (reference w/o coating) and PPy-coated fabrics were cut in pieces of approximately 2 cm^2^ (weight 0.1 g) and put on a steel stub for SEM analysis (ZEISS Gemini 500, Zeiss, Germany). The samples were sputter-coated with a 20 nm-thick gold layer in rarefied argon (20 Pa), using a Quorum SC7620 Sputter Coater (Quorum, Laughton, East Sussex, UK), with a current of 20 mA for 120 s.

The analyses were also performed on fabrics after extraction in artificial sweat (AS) at two different pHs (4.7 and 6.5). The extraction protocol in AS was performed according to ISO 10993-12 and is described in Section 2.6.

### 2.4. Washing Test

The coating stability and durability of PPy-coated fabrics were tested by a washing test in a wash-wheel (domestic laundering) according to ISO 105-C06. Fabric specimens of 10 × 4 cm, paired with adjacent fabrics of cotton and wool (Testfabrics Inc., West Pittston, PA, USA) with the same size, were plunged in 150 mL of a 4 g/L standard soap (ECE)–water solution. Washing cycles (number of total cycles, 25) were performed at 40 °C for 30 min, and after each cycle fabric samples were squeezed, rinsed in cold water for 10 min, and dried at RT. Additionally, color evaluations were performed to quantify the amount of PPy on the fabric surface by a Datacolor Spectraflash SF600 (Dietlikon, Rotkreuz, Switzerland) spectrophotometer, under CIE standard illuminant D65 and a 10° observer angle.

### 2.5. Antibacterial Efficacy Analysis

The antibacterial properties of PPy NPs were already reported in previous papers [19]. To confirm the action of the NPs newly synthesized for the purpose of this study, antibacterial tests were carried out on PPy dispersion at concentrations of 0.15, 0.25, 2.0, and 3.5 wt.% of PPy NPs against *Escherichia coli* with a procedure based on ASTM E2149-2013. The test bacterium culture was grown for 24 h in a suitable nutrient broth and then diluted to give a concentration of 1.5–3.0 × 10^5^ CFU/mL (inoculum). Next, 1.0 and 1.5 mL PPy dispersions at 4.1 wt.% were added in flasks containing 25 mL of the inoculum to obtain final PPy NPs concentrations of 0.15 and 0.25 wt.%, respectively. Then, 5.0 mL of the inoculum was added in flasks containing 5.0 mL of the PPy dispersion at 2.0 wt.% PPy NPs, and 1.0 mL of the inoculum was added in flasks containing 9 mL of the PPy dispersion at 3.5 wt.% PPy NPs. The contact time was 2 h at room temperature under stirring at 190 rpm. Then, 1.0 mL of the solutions was properly diluted in a buffer solution (pH 7) to have a maximum colony count of 150–300 CFU by plating 1.0 mL of diluted solutions in nutrient agar. Inoculated plates were incubated for 24 h at 37 °C, and finally the surviving colonies were counted. The antibacterial activity is expressed as % bacterial reduction of the micro-organisms after contact with the PPy NP dispersions compared to the number of bacterial cells that were originally present in the dilute inoculum solution. Tests were performed in duplicate, and results are the average of two separate counts.

### 2.6. Preparation of Textile Extracts

The extracts from textiles spray coated with PPy (about 3 g/m^2^) NPs and from uncoated textiles (reference) were obtained in artificial sweat (AS) as previously reported [20]. Briefly, fabrics were cut into pieces of 0.1 g (corresponding approximatively to 2 cm^2^) and put in borosilicate glass vials with 1 mL of AS. Sweat could have different pH in different body regions and be different due to age, gender, and physio-pathological conditions. Thus, two AS solutions were prepared at different pHs, 4.7 (ISO3160-2) and 6.5 (BS EN 18:11:2011), and extracts were incubated with both types of AS.

The chemicals used for the preparation of AS was purchased from Sigma Aldrich (Milano, Italy). Vials containing the fabrics in the two AS solutions were put on a shaker at 37 °C for 72 h (maximum time of exposure according to the ISO guidance). After the incubation, the extracts (about 800–900 µL) were recovered by squeezing the textiles with the help of a tweezer and stored at 4 °C until use. To determine whether the AS affected the release of PPy from textiles, a Datacolor experiment was performed as previously described (Section 2.4) on textiles after AS extraction at the two pH conditions. The analysis was performed in triplicate.

### 2.7. In Vitro Toxicological Analyses

#### 2.7.1. Cell Cultures’ Maintenance

An A549 cell line (ATCC^®^ CCL-185, American Type Culture Collection, Manassas, VA, USA) was cultured as previously described [21]. Balb/3T3 cells were maintained in DMEM high-glucose (4.5%) medium (Sigma Aldrich, Milano, Italy) supplemented with 10% fetal bovine serum (FBS, Gibco, Life Technologies, Monza, Italy) and 1% pen/strep (Euroclone, Pero, Italy), at 37 °C, 5% CO_2_. Co-cultures of epithelial lung cells (NCI-H441, ATCC^®^ HTB-174™) and endothelial pulmonary cells (HPMEC-ST1.6r), cultured for 12 days on opposite sides of Transwell inserts (pore size 0.4 µm) [22], were employed as 3D in vitro models of the air–blood barrier (ABB). Three-dimensional models of the reconstructed human epidermis (RhE) EpiDerm™ were purchased from MatTek corporation and maintained and used as previously described [23] and are summarized in Section 2.7.4.

#### 2.7.2. Cell Viability of Monocultures Exposed to NPs

For cell viability experiments, A549 cells (passages from 10 to 30) and Balb/3T3 cells (passage 2 from 15) were seeded on six-well plates (Corning^®^) at a concentration of 1.6 × 10^4^ and 3.2 × 10^4^ cell/cm^2^, respectively. After 24 h, cells were treated for 24 h with increasing concentration of PPy NPs (1, 10, 100, and 1000 µg/mL) by adding the particle suspension into the media (Opti-MEM + 1% FBS for A549 cells, DMEM + 1% FBS for Balb/3T3). Experiments were assessed in at least three independent replicates.

Cell viability was assessed through (MTT (3-(4,5-dimethylthiazol-2-yl)-2,5-diphenyltetrazolium bromide, Sigma Aldrich). At the end of exposure to PPy NPs, cells supernatants were removed, and cells were washed with phosphate-buffered saline (PBS). The MTT solution (0.3 mg/mL) was added in proper media (Opti-MEM or DMEM supplemented with 10% FBS) and incubated for 3 h. After the incubation time, cell media were removed and formazan crystals were solubilized with dimethyl sulfoxide (DMSO, Euroclone, Pero, Italy) and transferred in a 96-well plate for absorbance (Abs) measurement by means of a multiplate reader spectrophotometer (Infinite 200Pro, TECAN, Männedorf, Switzerland). Abs was read at 570 nm and at 690 nm (reference wavelength). Cell viability was measured as relative decrease compared to the Abs resulting from the control (100% of viable cells). Data are presented as mean ± standard error (SE) of at least three independent experiments.

#### 2.7.3. Co-Culture Model of the Air–Blood Barrier: Toxicity Endpoints

A 3D in vitro air–blood barrier (ABB), composed of human alveolar and lung endothelial cells grown on Transwell inserts, was used for additional inhalation toxicology studies. The ABB was cultured and differentiated according to [22,24] and then apically exposed to PPy NPs (10 and 100 µg/mL) for 24 h. After the treatments, three toxicological endpoints were evaluated:

− cell barrier integrity and functionality by the measurement of the transepithelial electrical resistance (TEER),
− cell viability by the colorimetric assay Alamar Blue test, and
− release of inflammatory cytokines, IL-8 and IL-6, by ELISA test.

Briefly, after 24 h exposure to PPy NPs, supernatants from both apical (epithelial cells) and basal (endothelial cells) compartments were collected, centrifuged at 1200 rpm for 6 min and stored at −80 °C for further ELISA analysis for the quantification of IL-6 and IL-8 cytokines (Novex, Life Technologies, Italy). Transwell inserts were rinsed twice with PBS and incubated with culture medium for 20–30 min at 37 °C for re-equilibration. Then, TEER was measured with an Epithelial Voltohmmeter (EVOM, World Precision Instruments, Berlin, Germany) equipped with an EndOhm 12 Chamber (World Precision Instruments, Berlin, Germany). The TEER of Transwell inserts w/o cells was measured and set as blank. TEER values (Ω × cm^2^) were calculated by subtracting the TEER of the blank (insert w/o cells). The surface area of the filter membrane was 1.12 cm^2^.

Data were expressed as the percentage ratio between TEER values on day 13 and TEER values on day 12 of co-culture. The mean ± SE of at least three independent experiments was presented. After TEER measurements, inserts were analyzed for cell viability through the Alamar Blue test (AlamarBlue^®^ Cell viability Reagent, Life Technologies, Monza, Italy), performed according to manufacturer’s instructions. The % cell viability related to the control samples (untreated cells) was calculated.

Three independent experiments were performed, and results were expressed as mean percent ± SEM of viable cells in comparison to the controls (untreated cells).

#### 2.7.4. The Reconstructed Human Epidermis Model EpiDerm™

The toxicity of NPs and NEPs was assessed on the reconstructed human epidermis model (RhE) EpiDerm™ (MatTek Corporation, Ashland, MA, USA), a 3D tissue model consisting of highly differentiated epidermal keratinocytes cultured on inserts. The RhE is recommended by OECD TG 431 [25] and 439 [26] for testing the corrosive and irritant potential of chemicals. At the arrival, tissues were recovered according to manufacturers’ instructions and then exposed to the NP suspensions or textile extracts according to the skin corrosion or irritation tests, respectively.

#### 2.7.5. Corrosion Test on EpiDerm™

The corrosion test (OECD TG 431) [25] on EpiDerm™ inserts was performed by directly exposing keratinocytes to 30 µL of NP suspensions in the apical compartment of the inserts, for 3 min and 1 h at different concentrations (1, 10, 100, and 1000 µg/mL). Fresh medium was added in the lower (basal) compartment of the inserts. PBS 1X was used as the negative control (NC) and KOH 8 M as the positive control (PC). After the exposure, inserts were processed according to the protocol, and then tissue viability was assessed through the MTT test. The absorbance of each sample was read by a multi-plate reader (Infinite 200 Pro, TECAN, Männedorf, Switzerland) at 570 nm. The tissue viability was calculated as a % with respect to the mean of the negative control (Abs sample/Abs control × 100). According to protocol, each experimental condition was analyzed in duplicate. The potential corrosiveness of the NPs was classified according the residual viability obtained after exposure and confronting the values with the Global Harmonized System (GHS) table adopted by the OECD guideline.

#### 2.7.6. Irritation Test on EpiDerm™

Irritation test (OECD TG 439) [26] was performed by exposing EpiDerm™ skin models for 18 h to 100 µL textile extracts (ISO/TC 194/WG 8). The textile extraction protocol was previously mentioned in Section 2.6. Negative control (NC, PBS 1X), positive controls (PC, SDS 1%), and reference materials (extracts of uncoated fabrics in AS were performed on dedicated inserts. After the incubation with extracts in AS at two different pH conditions (4.7 and 6.5), inserts were washed, and tissue viability was assessed through the MTT test as described for the corrosion test. The irritant potential of NPs was evaluated by calculating the % of tissue viability with respect to the mean of the negative control, as described above. For values below 50%, the substance is considered as an irritant of the 2nd category according to GHS classification, otherwise it is considered non-irritant.

Before incubation with MTT solution, supernatants form the basal compartment of the inserts were collected, centrifuged, and stored at −80 °C for ELISA analysis.

#### 2.7.7. Interleukin-8 Quantification from EpiDerm™ Model

Supernatants from the basal compartment of EpiDerm™ inserts were collected after exposure and analyzed for the quantification of IL-8, performed through IL-8 ELISA matched antibody pair kit (Invitrogen, Life Technologies, Monza, Italy) according to the manufacturer’s instructions. Data were expressed as pg/mL.

### 2.8. Statistical Analysis

Sigma Stat 3.2 software (Systat, Palo Alto, CA, USA) was used for statistical analyses (unpaired Student’s *t*-test). Values of *p* < 0.05 were considered statistically significant.

## 3. Results and Discussion

### 3.1. PPy NPs’ Characterization

**Figure 1:**
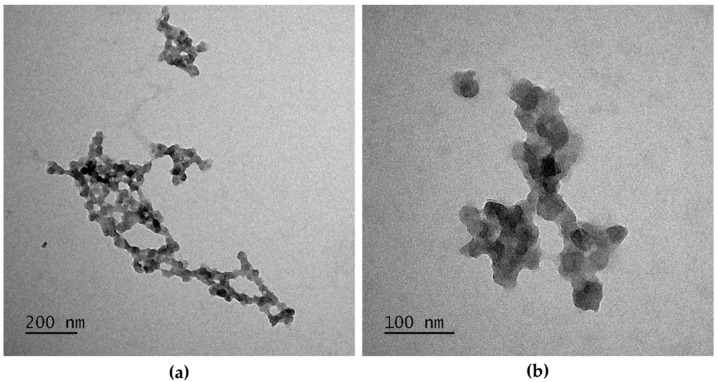
TEM images of PPy NPs. (**a**) PPy NPs aggregates (scale bar = 200 nm); (**b**) PPy NPs TEM images at higher magnification (scale bar = 100 nm).

Results from NP characterization show that PPy NPs tend to form aggregates that resemble a chain of irregular single NPs (single NP size < 50 nm) (Figure 1). Data from DLS analyses evidence the stability of PPy NPs in different aqueous media (mQ water and cell culture media and Opti-MEM and DMEM with 1% FBS) (Table 1). PPy NPs form smaller agglomerates of NPs in the culture cell media (252 and 241 nm in Opti-MEM and DMEM, respectively) compared to those in mQ water (331 nm). The PdI values were similar in the three different media and indicate that the NPs suspensions were monodispersed. The z-potential of PPy NPs is −55 mV, showing that PPy NPs are negatively charged due to the presence of PSS and that the NP suspensions have a good stability.

### 3.2. Characterization of PPy-Coated Textiles

The colorimetric quantification of PPy demonstrated that these NPs have good stability on cotton surface after washing tests, as considerable variations were not detected by this method, even after 25 washing cycles (Table 2). Likewise, PPy-coated fabrics after AS extraction show inconsiderable changes in the amount of PPy on cotton surface: 3.17 ± 0.23 and 3.26 ± 0.19 gPPy/m^2^ for pH values of 6.5 and 4.7, respectively. In general, NPs can deeply penetrate fabric surfaces, but, at the same time, they are easily released from clothes, and for this reason it could be necessary to apply compounds such as quaternary ammonium salts to enhance the surface attachment of NPs (i.e., Ag-NPs) [27,28]. However, PPy NPs remained solidly attached to the cotton surface, which gives clear evidence of the affinity that exists between cotton fibers and PPy NPs, as previously reported in literature [29].

**Figure 2:**
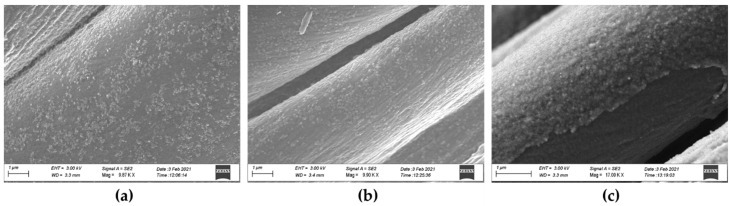
HR-SEM of PPy-coated cotton fabrics. (**a**) PPy-coated fabrics with PPy NPs before extraction in artificial sweat (AS); (**b**) PPy-coated fabrics after 72 h extraction in AS at pH 4.7; (**c**) PPy-coated fabrics after 72 h extraction in AS at pH 6.5.

The PPy-coated cotton fabrics were also characterized by SEM in order to evaluate the quality of the coating. The analysis was performed on reference cotton fabrics (not coated) and on PPy-coated fabrics (about 3 g PPy/m^2^), before and after artificial sweat extraction. Data showed that the cotton fibers are quite homogenously coated with PPy (Figure 2) and that the extraction in AS at different pHs seems not to influence the coating, since PPy NPs are still present on the fabric’s fibers. In some spots, the coating seemed discontinuous but that seems more attributable to the abrasion of the fibers than to the extraction in AS (data not shown).

It is well known that the deposition of NPs on textiles materials can be accompanied by the release of these functional NMs, which give rises to an increasing concern about their applications and safety. Nevertheless, PPy NPs have been reported to have excellent stability on cotton surfaces (after washing or AS extraction), diminishing the risk of any possible diffusion into the interstitium. These results are outstanding in comparison with results reported in literatures for metal NPs (Ag-NPs) or metal oxide NPs (TiO_2_-NPs) [30,31,32].

### 3.3. Antibacterial Efficiency of PPy

**Figure 3:**
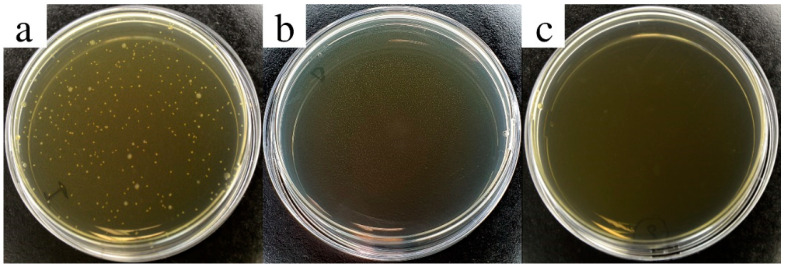
Antibacterial test. *Escherichia coli* colonies grown after incubation in the dilute inoculum bacterial solution (**a**) and in PPy NP concentrations of 2.0 wt.% (**b**) and 3.5 wt.% (**c**).

Antibacterial tests were performed against *Escherichia coli* on PPy NP dispersions in water at final NP concentrations of 2.0 and 3.5 wt.%. Figure 3 shows the pictures of Petri dishes after 24 h of incubation. In particular, the Figure 3a shows the bacterial growth related to the bacterial inoculum not contacted with the PPy NPs (as control). A large number of colonies had grown on the culture medium. Figure 3b,c shows the Petri dishes of the bacterial inoculum after a contact time of 2 h at room temperature under stirring with PPy NPs at final concentrations of 2.0 and 3.5 wt.%, respectively. No colony had grown, hence no bacteria survived the contact with PPy NPs in both concentrations. Therefore, the resulting bacterial reduction percentage was 100%. When the concentration of PPy NPs was reduced to 0.15 and 0.25 wt.%, the bacterial reduction considerably decreased (0% and 2%, respectively). These results have demonstrated that the biocidal activity of PPy NPs strongly depends on concentration in solution, and, by the same token, the antibacterial properties of PPy-coated cotton rely on the amount of PPy NPs deposited on the fabrics.

### 3.4. Toxicity of PPy NPs on In Vitro Models

#### 3.4.1. Cell Viability of A549 Lung Cells and Balb/3T3 Fibroblast Monocultures

**Figure 4:**
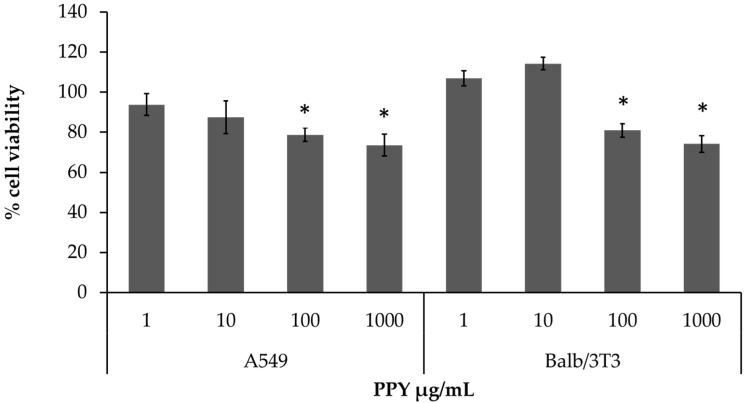
Cell viability. The MTT test was performed on A549 and Balb/3T3 cells after exposure for 24 h to increasing concentrations (1, 10, 100, and 1000 µg/mL) of PPy NPs. Data represent the mean ± SE of at least three independent experiments. * Statistically different compared to control samples. *p* < 0.05. One-way ANOVA + Bonferroni’s.

Data from MTT test performed on two different cell lines, A549 and Balb/3T3 cells, after exposure to increasing concentrations of PPy NPs, showed that the tested NPs induced a significant decrease of cell viability starting from the concentration of 100 µg/mL (Figure 4). Previous data from Kim at al. [33] showed that the capability of PPy NPs to induce cytotoxicity is related to the NPs’ size and to the cell type. Lung fibroblasts (IMR90) are less sensitive to PPy NPs in comparison with immune cells (J774A.1). Here, epithelial cells and fibroblasts seemed to have similar responses, since a reduction of cell viability was observed in both cell types only after a concentration of 100 µg/mL. PPy NPs form a chain-like structure composed of single NPs having a size of 40–60 nm. Data from the cell viability test are in accordance with previous works in which PPy NPs of similar size were biocompatible with different mammalian cells [33]. These data confirmed that PPy NPs at low concentration are biocompatible, while at high concentrations they became cytotoxic to A549 and Balb/3T3 cells, as also was evidenced for other cells lines such as Jurkat, MEF, and MH-22A cells [12]; human lung fibroblasts (IMR90); and mouse alveolar macrophages (J774A.1) [33]. The cytotoxicity of PPy NPs, which is dose and size dependent, it is mostly due to NP uptake by endocytosis and the consequent induction of ROS generation, activation of the apoptosis/necrosis pathways, and, thus, cytotoxic effects, by proliferation inhibition [12].

#### 3.4.2. Effects of PPy NPs on In Vitro Air–Blood Barrier

**Figure 5:**
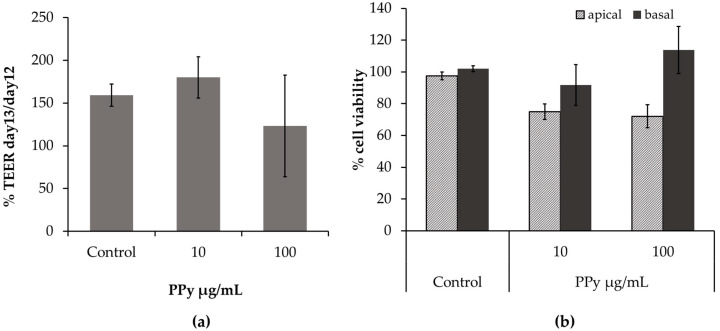
In vitro ABB model integrity. (**a**) TEER of the ABB model after exposure for 24 h to PPy NPs (10 and 100 µg/mL). TEER values were expressed as ratio % between TEER values on day 13 and values on day 12. (**b**) Cell viability of the upper (apical) and lower (basal) compartments of the inserts after exposure to PPy NPs was assessed through the Alamar Blue assay. Data are expressed as relative decrease compared to control (untreated cells), considered as 100% of viable cells. Results represent the mean ± SE of three independent experiments (*n* = 3).

**Figure 6:**
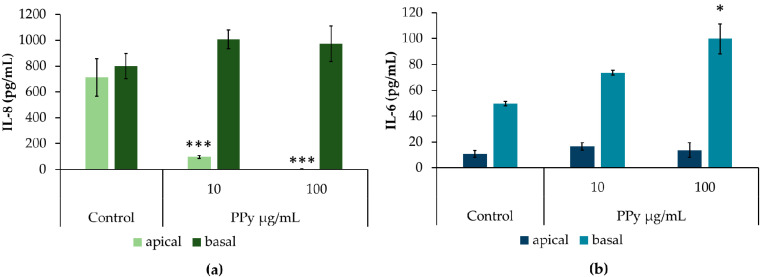
Cytokine release. IL-8 (**a**) and IL-6 (**b**) release from both upper (apical) and lower (basal) compartments of the inserts apically exposed for 24 h to PPy (10 and 100 µg/mL). Data were expressed in pg/mL and represented as mean ± SE of three independent experiments (*n* = 3). * *p* < 0.05; *** *p* < 0.001. Significantly different from the respective control.

The functionality of the in vitro model of the ABB after exposure to PPy NPs was determined by the evaluation of three endpoints: epithelial resistance (TEER) (Figure 5a), cell viability (Alamar Blue test) (Figure 5b), and release of inflammatory mediators IL-8 (Figure 6a) and IL-6 (Figure 6b) (ELISA test) from both epithelial and endothelial compartments. Data from TEER measurements showed that at the concentration of 100 µg/mL, apical exposure to PPy NPs reduced the tightness of the ABB, although not significantly. Data from Alamar Blue revealed that the exposure to PPy induced a slight, though not significant (*p* > 0.05), reduction of cell viability at the apical (epithelial) compartment of the inserts, but the viability at the basal (endothelial) side seemed not affected by the apical treatment, with consequent maintenance of the barrier integrity and functionality at the tested concentrations. To our knowledge, there are no data in the literature regarding the effects of polymeric PPy NPs on a 3D model of the lung that more closely resemble the ABB with respect to classic cellular monocultures.

The release of inflammatory mediators, such as interleukins (ILs) IL-8 and IL-6, is another key parameter that should be evaluated after the epithelial barrier exposure, in order to understand the inflammatory local effects (at the respiratory epithelium) and possible systemic effects by the quantification of ILs from the basal compartment of the barrier. Data showed that IL-8 release was significantly decreased (*p* < 0.001) at the apical level after treatment with increasing concentrations of PPy NPs. However, this effect seems to be due to the depletion of IL-8 from the supernatants. NPs are discharged from the supernatants by centrifugation before their storage until analyses. If IL-8 remains linked to the PPy NPs, with a sort of grafting (and consequently depleting) effect, it could be removed after centrifugation.

Interestingly, at the higher concentration tested (100 µg/mL) a statistically significant increased release of IL-6 (*p* < 0.05) from the endothelial compartment was observed, suggesting that the apical exposure to PPy stimulated endothelial cells’ secretion of a pro-inflammatory mediator that is recognized to be involved in the recruitment of monocytes in response to potentially harmful compounds.

#### 3.4.3. Toxicity of PPy NPs and Textile Extracts on Reconstructed Human Epidermis

**Figure 7:**
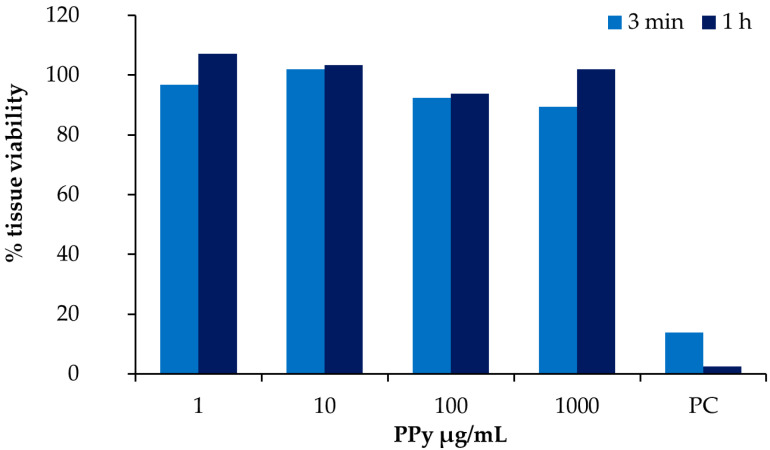
Corrosion skin test. EpiDerm™ was exposed for 3 min and 1 h to PPy NPs (1, 10, 100, and 1000 µg/mL) and the % of tissue viability was assessed through MTT test. Potassium hydroxide (KOH) at the concentration of 8 M was used as a positive control (PC), and PBS 1X as a negative control. The bars represent the mean values of two repetitions (N = 2) for each test conditions.

Skin corrosion is an irreparable damage that causes loss of function of the epidermidis. Thus, corrosiveness is an extremely important endpoint during the safety assessment of a substance. The potential corrosiveness of PPy NPs was evaluated through the direct exposure of EpiDerm™ skin models to NP water suspensions at different concentrations and times of exposure (3 min and 1 h), following the OECD TG 431. Data in Figure 7 show that, even at the highest concentrations of exposure (1000 µg/mL), PPy NPs did not reduced the tissue viability of the skin models. The tissue viability was >50% and >15% at 3 min and 1 h respectively, thus PPy NPs are not corrosive for the skin according to the classification criteria of the Globally Harmonized System (GHS).

**Figure 8:**
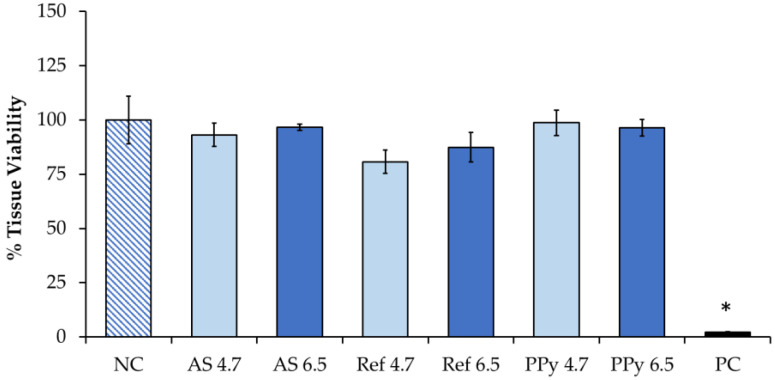
Irritation skin test. EpiDerm™ was exposed to textile extracts from NP-coated (PPy 4.7 and PPy 6.7) and reference textiles (Ref 4.7 and Ref 6.7). Unexposed cells (negative cells, NC) were incubated with PBS 1X and SDS 1% was used as the positive control (PC). Data represent the % of tissue viability evaluated through MTT test after 18 h of exposure. Bars represent the mean ± SE of three replicates (N = 3). * *p* < 0.001 vs. NC (Student’s *t* test).

On the other hand, skin irritation causes a reversible impairment of the epidermis, and it can occur at the skin after exposure to tested compounds. The irritation test was performed by exposing skin tissues to NEP (PPy-coated fabrics) extracts, which is a more physiological and realistic exposure condition that mimics a consumer end-use scenario. Figure 8 shows that at both pH 4.7 and pH 6.5 PPy-coated fabrics are not irritants, since the tissue viability percentage was above the 50% and no statistically relevant differences (*p* > 0.05) were observed among control samples (NC, exposed to PBS 1X) and samples exposed to textile extracts or AS or reference (uncoated textiles) samples at both pHs. The only statistically significant difference was observed after exposure to SDS 1% (*p* < 0.001), which is the positive control for this type of test according to the MatTek protocol. These data on skin irritation are very promising, since metal-based NPs or MeO-NP-coated textiles are known to be hazardous in some cases, due to the release of metal ions in acidic AS [20,27]. Data from SEM and Datacolor analyses suggest that PPy remains attached to the cotton fibers, with no significant leaching in the AS at both pHs. It has to be taken into account that PPy NPs are also used in different applications for reducing stenosis and inflammation in biomedical devices [34].

**Figure 9:**
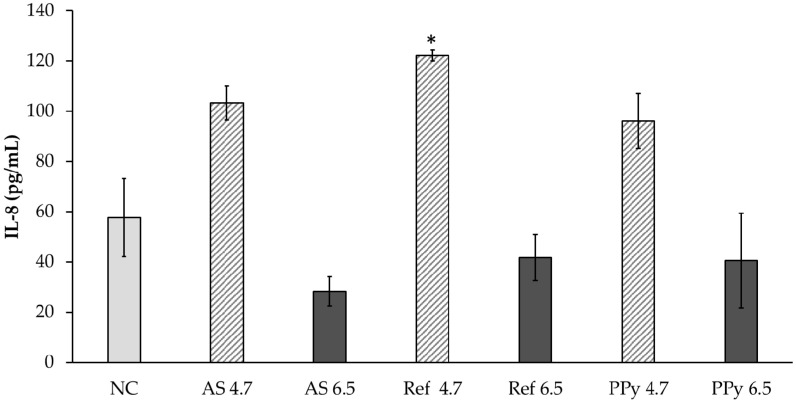
Release of IL-8 after 18 h of exposure of EpiDerm ™ to textile extracts obtained from PPy-coated textiles extracted for 72 h at 37 °C in AS at pH 4.7 and 6.5. NC: negative control (PBS 1X); AS: artificial sweat solution; Ref: reference material (fabric without NPs). * *p* < 0.05; One-way ANOVA + Bonferroni’s, statistically different compared to negative control sample.

The release/leaching of NMs from textiles could also cause a release of inflammatory mediators that it is an index of irritation of the skin. Among the interleukins, IL-8 is one of the precursors of the inflammatory response linked to skin irritation [35]. IL-8 release was measured in the basal compartment of the inserts after the apical exposure to PPy-coated textile extracts. Data (Figure 9) showed that increased levels of IL-8 are appreciable at acidic pH conditions (Ref 4.7, *p* < 0.05) of the AS rather than due to the presence of PPy NPs, evidencing that the acidic pH itself seems to be associated with an inflammatory response of skin cells. At pH 6.5, no differences (*p* > 0.05) between treated samples and the control were observed.

No corrosion or irritation was observed in the tissues exposed to PPy suspensions (Figure 7) or extracts from coated textiles (Figure 8), respectively, allowing us to retain this material as safe toward the epidermis for application in coating textiles. This evidence is also supported by the literature, in which several papers show the efficacy and biocompatibility of PPy NPs even in other area of operation, such as photothermal therapy [36] and wound skin care [2].

## 4. Conclusions

In this paper, the safety of PPy NPs and PPy-coated cotton fabrics, developed to confer antibacterial properties, has been assessed considering two important steps of possible NM release and human exposure during the life cycle: (i) NP inhalation exposure during the coating process and (ii) the skin contact during the use of the final product. Thus, in vitro toxicity studies have been performed using different models, including monoculture and 3D in vitro co-culture systems. Data showed that NPs and coated fabrics are safe for reconstructed human epidermidis. The respiratory tissue seems to be more affected by PPy NPs but at very high exposure concentrations that are unlikely to correspond to real exposure scenarios. Anyway, the potential to induce inflammatory response at the respiratory barrier level, suggests precautions for exposed workers. Altogether, this evidence shows that PPy-based NMs are promising candidates for future application in textiles or other antibacterial nanotechnologies, mainly thanks to their stability and biocompatibility to skin, which is the main target organ of surface coating NMs. Moreover, the data obtained give promising insights for the future research of PPy NPs for the spray coating of textiles with antiviral properties, also against SARS-Cov-2 as recently reported [37]. It has been indeed reported that conductive polymers are capable of binding different strains of viruses, including avian and influenza A and B human viruses, and that the functionalization of polymers with silver would increase the efficiency of virus adsorption, and almost complete the removal of viruses from aqueous media [38]. Of course, further investigations are required to improve the efficacy and industrial implementation of these NMs, as well as the possible adverse effects, especially after inhalation, over prolonged periods of exposure to low NP doses.

## Figures and Tables

**Table 1:** Z-average (nm) and polydispersity index (PdI) of 100 µg/mL PPy NPs measured by dynamic light scattering (DLS) and in two different media (mQ water and culture media). Means ± SD of three replicates.

NPs	Medium	Z-Average (nm) ± SD	PdI ± SD
PPy	mQ water	331 ± 0.1	0.226 ± 0.013
	DMEM 1% FBS	241 ± 2.1	0.235 ± 0.025
	Opti-MEM 1% FBS	252 ± 8.3	0.232 ± 0.026

**Table 2:** PPy amount on cotton fabric measured by colorimetric analysis after different washing cycles (ISO 150).

ISO 150 Washing Cycles	g PPy/m^2^
0	3.07 ± 0.21
1	3.13 ± 0.10
5	3.04 ± 0.10
10	3.09 ± 0.11
25	2.97 ± 0.12

## Data Availability

Data is contained within the article.
